# Mindfulness-based intervention for patients with generalized anxiety disorder: a randomized controlled trial

**DOI:** 10.3389/fpsyt.2026.1810049

**Published:** 2026-04-23

**Authors:** Cancan Zhang, Jin Zhang, Lei Sun

**Affiliations:** Psychiatry department, Tianjin Anding Hospital, Tianjin, China

**Keywords:** generalized anxiety disorder, intervention, linear mixed-effects model, mindfulness, randomized controlled trial

## Abstract

**Background:**

Despite growing interest in mindfulness-based interventions (MBI) for Generalized Anxiety Disorder (GAD), robust evidence for their adjunctive use alongside first-line pharmacotherapy is scarce. This study aimed to evaluate the efficacy of MBI as adjunct to pharmacotherapy in adults with GAD, assessing multidimensional outcomes.

**Methods:**

In this randomized controlled trial, 106 adults diagnosed with DSM-5 GAD and on stable pharmacotherapy were randomly assigned to intervention group or control group (n=53). The intervention group received an 8-week, group-based MBI adapted from Mindfulness-Based Cognitive Therapy in addition to their ongoing pharmacotherapy. The control group received pharmacotherapy plus an active control condition consisting of structured psychoeducation (n=53). The primary outcome was the change in clinician-rated anxiety severity on the Hamilton Anxiety Rating Scale (HAMA). Secondary outcomes included self-reported anxiety (Self-Rating Anxiety Scale, SAS), mindfulness skills (Five Facet Mindfulness Questionnaire, FFMQ), sleep quality (Pittsburgh Sleep Quality Index, PSQI), negative cognitive bias (Negative Cognitive Processing Bias Questionnaire, NCPBQ), and psychosocial functioning (Global Assessment of Functioning-Modified, GAF-M). Linear mixed-effects model was used for analysis.

**Results:**

The intervention group demonstrated a significantly greater reduction in HAMA scores post-intervention compared to the control group (18.6 vs. 22.5; *P* < 0.05), with a significant group × time interaction (Estimate = -3.68, *P* < 0.05). Significant between-group improvements favoring the intervention group were also confirmed across all secondary outcomes: SAS (Estimate = -4.29, *P* < 0.05), FFMQ (Estimate = 20.25, *P* < 0.05), PSQI (Estimate = -2.36, *P* < 0.05), NCPBQ (Estimate = -6.75, *P* < 0.05), and GAF-M (Estimate = 6.98, *P* < 0.05).

**Conclusion:**

An adjunctive, 8-week MBI significantly reduces anxiety and enhances mindfulness, sleep, cognitive function, and psychosocial well-being in medicated GAD patients. These findings support integrating structured MBI into standard care to optimize functional recovery alongside symptom management.

**Trial registration:**

The study was registered prior to conducting the research in the Chinese Clinical Trial Registry (https://www.chictr.org.cn) on September 26, 2024 with the trial identification number ChiCTR2400090284

## 1 Introduction

Generalized Anxiety Disorder (GAD) is a prevalent and debilitating psychiatric condition characterized by persistent, excessive worry and difficult-to-control anxiety regarding everyday matters. Its clinical presentation encompasses both psychological symptoms, such as restlessness, irritability, and impaired concentration (1), and somatic complaints, including fatigue, muscle tension, and sleep disturbances (2). With a global lifetime prevalence of approximately 3.7%, GAD imposes a substantial worldwide disease burden, marked by significant functional impairment and diminished quality of life (3). In China, GAD ranks among the most common mental health disorders, with a reported 12-month prevalence of 3.5%, contributing significantly to the country’s mental health burden (4). The illness typically follows a chronic and relapsing-remitting course, frequently resulting in impairments across occupational, social, and personal domains while concurrently elevating the risk for comorbid conditions, such as other anxiety disorders, major depressive disorder, and substance use disorders (5).

First-line management for GAD involves pharmacotherapy and psychotherapy (6). Pharmacological interventions primarily include selective serotonin reuptake inhibitors (SSRIs) and serotonin-norepinephrine reuptake inhibitors (SNRIs), with benzodiazepines reserved for short-term symptom relief. Among psychotherapeutic approaches, cognitive-behavioral therapy (CBT) is the most established. Although these treatments are empirically validated, they present notable limitations. Pharmacotherapy is often associated with side effects, variable individual response, and potential dependence. A particularly significant clinical challenge is the high risk linked to treatment discontinuation. Cessation of antidepressants can precipitate a withdrawal syndrome with considerable incidence and, more critically, exposes patients to a substantial risk of symptom relapse (7). Systematic reviews indicate that the relapse rate for GAD patients after discontinuing successful pharmacotherapy is substantially elevated, with hazard ratios for relapse ranging from 0.12 to 0.58 compared to continued treatment (8). Conversely, the widespread dissemination of CBT is hindered by barriers such as high cost, limited access to trained therapists, patient preferences, and the significant cognitive demand placed on individuals, which can restrict adherence and implementation (9). For instance, in many regions, including parts of China, the availability of trained CBT therapists is scarce, leading to long wait times and leaving a large treatment gap. These constraints highlight a clear need for the development, evaluation, and integration of complementary therapeutic strategies that are more accessible and sustainable.

In recent decades, mindfulness-based interventions (MBI) have emerged as a promising adjunctive treatment paradigm for anxiety disorders (10). Clinically operationalized through standardized protocols such as Mindfulness-Based Stress Reduction (MBSR) and Mindfulness-Based Cognitive Therapy (MBCT), MBI are grounded in the intentional cultivation of present-moment awareness and a non-judgmental attitude (2). The theoretical framework posits that by fostering a metacognitive, decentered stance toward internal experiences, individuals can disrupt habitual patterns of maladaptive cognitive-emotional reactivity, a mechanism supported by emerging evidence (10). This shift from a reactive doing mode to an accepting being mode is theorized to mitigate negative cognitive biases, enhance emotion regulation, and reduce experiential avoidance, thereby targeting core psychopathological mechanisms of GAD. Empirical evidence, including meta-analytic findings, reports small to moderate effect sizes for anxiety symptom reduction across diverse populations (11). Preliminary GAD-specific studies show that group-based 8 to 10-week MBI can yield sustained benefits (12). Neuroscientific research further suggests that mindfulness training may induce neuroplastic adaptations in brain regions critical for attention and emotion processing, such as the prefrontal cortex and amygdala, providing a potential neurobiological substrate for its therapeutic effects (13).

However, several methodological and conceptual limitations in the current literature necessitate further rigorous investigation. Common shortcomings include studies with small sample sizes, reliance on waitlist control conditions, heterogeneous intervention protocols, and insufficient probing of therapeutic mechanisms. Despite the widespread use of pharmacotherapy as a first-line treatment, there is a scarcity of research explicitly testing the additive or synergistic benefits of combining structured MBI with stable, optimized pharmacotherapy for GAD; The research focus has predominantly been on symptom reduction, leaving the impact of MBI on broader functional recovery inadequately examined; Few studies have incorporated mechanism-oriented outcomes, such as mindfulness skill acquisition and changes in cognitive biases, to elucidate the causal pathways of therapeutic change. In addition, a particularly salient limitation is the scarcity of research explicitly testing the additive or synergistic benefits of combining mindfulness training with first-line pharmacological treatments for GAD. This deficit is of direct clinical relevance, given that most patients with moderate-to-severe GAD are managed with pharmacotherapy.

Therefore, this study aims to address these limitations by conducting a randomized controlled trial to systematically evaluate the adjunctive efficacy of a structured MBI for patients diagnosed with GAD who were concurrently receiving pharmacotherapy. The novelty of this study lies in its rigorous design, which includes an active control group, a focus on multidimensional outcomes encompassing functional recovery and mechanistic variables, and the examination of the intervention’s additive value to standard pharmacotherapy. The trial aims to generate high-quality evidence to inform strategies for optimizing both clinical and functional outcomes in GAD and to contribute to the practical foundation for culturally contextualized mindfulness interventions.

## 2 Methods

### 2.1 Study design

This study was a randomized controlled trial conducted at Tianjin Anding Hospital from January 2023 to December 2025. The study received ethical approval from the Tianjin Anding Hospital’s ethics committee (No. 2023-64) and was registered in a publicly accessible clinical trial registry (Trial Registration: ChiCTR2400090284). The conduct and reporting of this trial adhered to the Consolidated Standards of Reporting Trials (CONSORT) guidelines.

### 2.2 Participants

Participants were recruited through outpatient clinics at Tianjin Anding Hospital via consecutive sampling. The sample size was calculated *a priori* to ensure adequate power to detect a between-group difference in the primary outcome, defined as the mean change in the Hamilton Anxiety Rating Scale (HAMA) score from baseline to post-intervention. The target mean difference (δ) was set at 4.5 points, and the pooled standard deviation (σ) was estimated to be 7.5 points, based on data from prior clinical trials in comparable populations (11). For a two-tailed test with an alpha (α) level of 0.05 and a statistical power (1-β) of 80%, the required sample size per group (n) was calculated using the standard formula for comparing two independent means:


$$n=2{\left[(Z_{\alpha /2}+Z_{\beta })/(\delta /\sigma )\right]}^{2}$$

**Figure 1:**
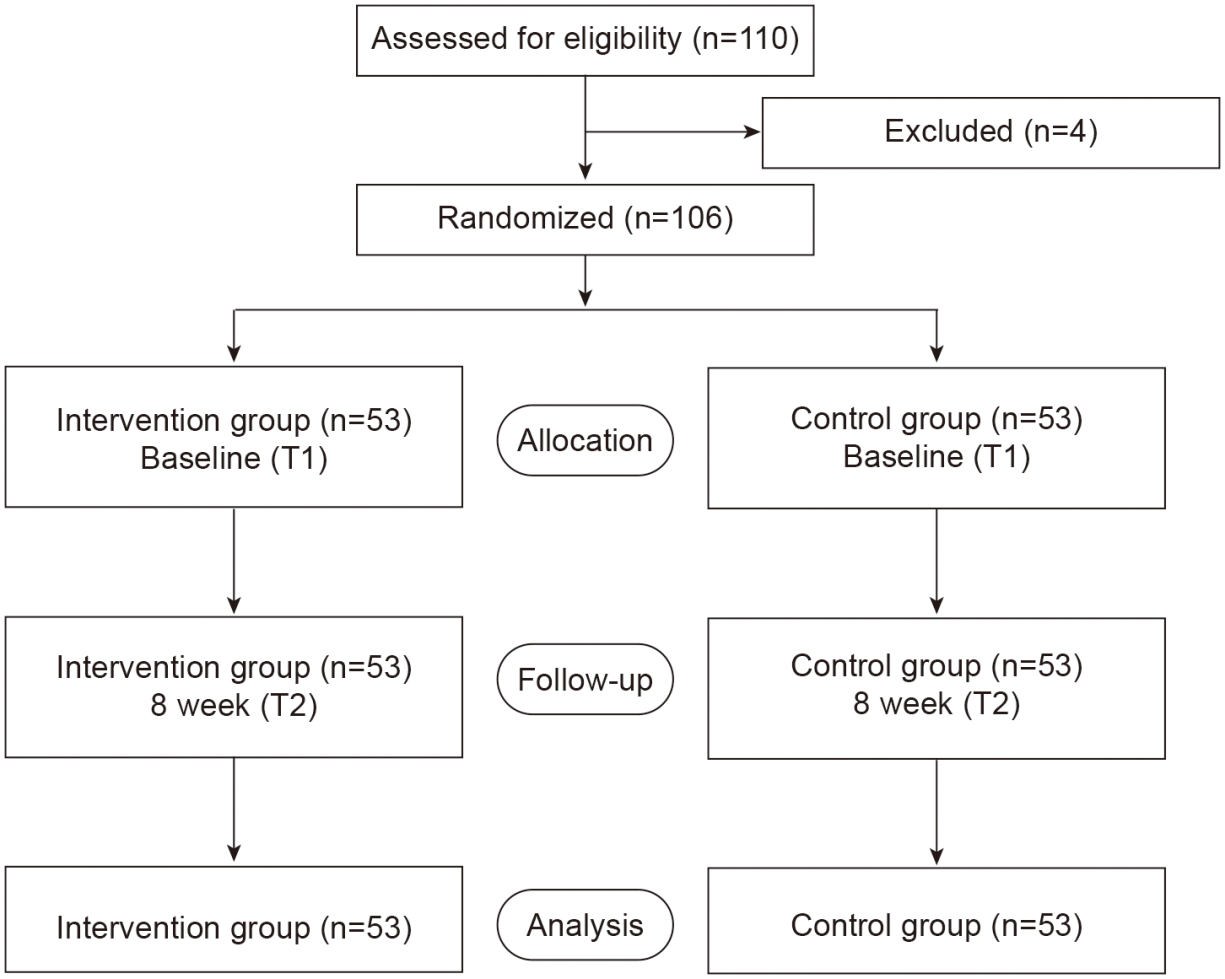
Study flow diagram.

The calculation indicated a requirement of 44 participants per group. To account for an anticipated attrition rate of approximately 20%, the final recruitment target was set at 55 participants per group, for a total sample size of 110. Inclusion Criteria (1): Aged between 18 and 65 years (2); A primary diagnosis of GAD meeting DSM-5 criteria; (3) A stable, optimized regimen of first-line pharmacotherapy for at least 6 weeks prior to enrollment, with no planned dosage changes during the trial; (4) Willingness and ability to provide written informed consent and commit to the intervention schedule; (5) A baseline HAMA total score ≥ 20, indicating moderate-to-severe symptom severity; Exclusion Criteria: (1) A current comorbid diagnosis of schizophrenia, bipolar disorder, major depressive disorder with active suicidal ideation, or obsessive-compulsive disorder; (2) Active substance abuse or dependence disorder within the preceding 6 months; (3) Severe cognitive impairment, significant neurological disorder, or any unstable medical condition that could interfere with participation; (4) Regular mindfulness or meditation practice (defined as > once per week) in the year prior to enrollment; (5) Concurrent participation in any other structured psychotherapy program; (6) Inability to comprehend the study procedures or complete the assessment questionnaires. Finally, the 4 were excluded (3 did not meet inclusion criteria, 1 declined to participate), leaving 106 patients who were randomized (see **Figure 1**). Participants were randomly allocated in a 1:1 ratio to either the intervention group or the control group using a random number table. A random number sequence was generated by an independent statistician not involved in the trial’s conduct. Allocation concealment was ensured by using sequentially numbered, opaque, sealed envelopes. Due to the nature of the intervention, blinding of participants and therapists was not possible. However, outcome assessors and the statistician performing the primary analyses were kept blinded to group allocation.

### 2.3 Intervention

Participants randomized to the control group received enhanced treatment-as-usual (ETAU). This intervention comprised two core components: ongoing stable pharmacotherapy and structured basic health services. Pharmacotherapy followed standardized first-line protocols for GAD, primarily involving SSRIs or SNRIs, as managed by the participant’s treating psychiatrist. The enhanced, structured component consisted of eight weekly, manualized group sessions, each lasting approximately 60 minutes. These sessions provided general mental health education and supportive monitoring, covering topics such as anxiety psychoeducation, sleep hygiene, stress management, and lifestyle advice. It is critical to note that these sessions deliberately excluded any instruction in mindfulness training, meditation practices, or core CBT techniques. This design ensured that the control condition adequately accounted for non-specific therapeutic factors, including time, professional attention, and group dynamics, thereby allowing the specific effects of the mindfulness component to be isolated in the subsequent analysis.

Participants allocated to the intervention group received pharmacotherapy, managed on identical principles to the control group, plus an 8-week structured MBI. The MBI protocol was adapted from the standardized MBCT framework, with specific modifications to target the core psychopathology of GAD, such as pervasive worry and somatic tension. The program included eight weekly, facilitator-led group sessions of 2 to 2.5 hours each. Sessions sequentially introduced core mindfulness practices and integrated psychoeducation focused on recognizing and relating differently to anxious thoughts and bodily sensations through a stance of decentering and acceptance. To support skill acquisition and integration, participants were assigned daily formal and informal mindfulness practices, targeting 30 to 45 minutes per day, which were guided by audio recordings and practice logs. The overarching therapeutic aim of the intervention was to cultivate present-moment awareness and non-judgmental acceptance, thereby theoretically disrupting the characteristic cycle of maladaptive cognitive-emotional reactivity in GAD. All intervention group facilitators were licensed clinical psychologists with at least 3 years of experience in leading mindfulness-based programs. Both had completed intensive 5-day teacher training in MBCT and maintained their own personal mindfulness practice. They adhered to a detailed treatment manual to ensure consistency and treatment fidelity across all intervention groups.

For all participants in both groups, a stable and optimized medication regimen was a prerequisite, requiring at least 6 weeks of consistent treatment prior to randomization. Treating psychiatrists were requested to avoid altering medication types or dosages during the 8-week intervention period unless clinically imperative. Detailed information on medication classes (SSRIs and SNRIs) and mean dosages (fluoxetine-equivalent doses) was recorded at baseline and post-intervention to confirm stability and assess any potential pharmacological imbalance between groups.

### 2.4 Data collection

Data were collected at two predefined time points: at baseline (T0, prior to randomization and the initiation of any study intervention) and immediately post-intervention (T1, within one week after completing the 8-week program). At baseline, comprehensive demographic and clinical information was obtained through a structured case report form. This included age, gender, body mass index (BMI), educational, marital status, duration of GAD, and chronic medical history (diabetes, hypertension). In addition, we have collected information on medication class (SSRIs/SNRIs) and fluoxetine-equivalent dose.

The primary outcome was the HAMA, employed as the primary outcome measure to assess the severity of anxiety symptoms (14). This scale is a widely used clinician-administered instrument for evaluating anxiety severity. The HAMA consists of 14 items that assess both psychological and somatic symptoms of anxiety. Each item is rated on a 5-point scale from 0 to 4, with the total score reflecting the overall severity of anxiety.

Secondary outcomes were measured using validated self-report instruments. The Self-Rating Anxiety Scale (SAS) was used to evaluate the patient-reported severity of anxiety symptoms (15). The SAS is a 20-item self-report instrument that has been extensively used in Chinese populations and has demonstrated acceptable psychometric properties. The Five Facet Mindfulness Questionnaire (FFMQ) was administered to assess the development of mindfulness skills (16). The FFMQ is a core instrument for measuring trait mindfulness, encompassing five dimensions: observing, describing, acting with awareness, non-judging, and non-reactivity. The Pittsburgh Sleep Quality Index (PSQI) was utilized to evaluate subjective sleep quality (17). The PSQI is a 19-item self-report instrument designed to measure sleep quality and disturbances over the past month. It yields seven component scores and one global score, with a global score of ≥5 typically indicating poor sleep quality. The Negative Cognitive Processing Bias Questionnaire (NCPBQ) was employed to assess maladaptive cognitive patterns associated with anxiety (18). This questionnaire is designed to measure an individual’s tendency to prioritize negative stimuli during information processing. The modified version of the Global Assessment of Functioning-Modified (GAF-M) was used to evaluate the patient’s overall level of psychosocial functioning (19). This scale, derived from the Axis V assessment in the DSM system, was revised to provide a more reliable assessment of functional status. All scales have been previously validated in Chinese populations, demonstrating good psychometric properties. In the current sample, internal consistency (Cronbach’s α) for these measures ranged from 0.79 to 0.88 at baseline, indicating acceptable reliability.

All assessments were conducted by research personnel who were blinded to the participants’ group allocation to minimize assessment bias.

### 2.5 Statistical analysis

Statistical analyses were performed using SPSS (version 26.0). Categorical variables are presented as frequencies and percentages (n, %) and were compared using the chi-square and Fisher’s exact test. Continuous variables were tested for normality with the Shapiro-Wilk test. Normally distributed data are expressed as mean ± standard deviation (SD) and compared using the independent samples t-test. To analyze the longitudinal outcomes across the two assessment time points (T1, T2), a linear mixed-effects model (LMM) was employed as the primary analytical framework (20). Missing data were handled using the LMM. This model was chosen for its flexibility in handling repeated measures data and ability to account for within-subject correlation. The fixed effects in the model included time (as a categorical factor), group (intervention/control), and the time-by-group interaction. From this model, we extracted the group × time interaction estimates, which represent the mean difference in change from baseline to post-intervention between the two groups, along with their 95% confidence intervals (CIs). Standardized effect sizes (Cohen’s d) were calculated for the primary between-group comparisons by dividing the mean difference by the pooled baseline standard deviation. All analyses were conducted on an intention-to-treat (ITT) basis, including all randomized participants. For all analyses, a two-sided *P*-value < 0.05 was considered statistically significant.

## 3 Results

### 3.1 Characteristics of patients

A total of 106 patients were randomized into the intervention and control groups (53 per group). As shown in **Table 1**, the two groups were comparable on all baseline demographic and clinical characteristics, including age, gender, BMI, education, marital status, duration of GAD, and prevalence of diabetes and hypertension, with no statistically significant differences observed (all *P* > 0.05). Furthermore, the types of pharmacotherapy and mean dosages were similar between the two groups at baseline and remained stable throughout the trial (all *P* > 0.05). This indicates that the randomization procedure successfully produced well-balanced groups.

**Table 1:** Characteristics of patients.

Variables	Intervention group (n=53)	Control group (n=53)	*t*/*χ*^2^	*P*
Age, years	38.0 ± 14.2	35.6 ± 12.9	0.909	0.365
BMI, kg/m²	24.2 ± 4.7	24.0± 4.5	0.230	0.819
Gender			0.358	0.550
Female	22 (41.5)	19 (35.8)		
Male	31 (58.5)	34 (64.2)		
Education level			0.162	0.922
High school or below	10 (18.9)	9 (17.0)		
College/University	20 (37.7)	19 (35.8)		
Postgraduate	23 (43.4)	25 (47.2)		
Marital status			–	0.689
Single	24 (45.3)	24 (45.3)		
Married/Cohabiting	27 (50.9)	25 (47.2)		
Divorced/Widowed	2 (3.8)	4 (7.5)		
Duration of GAD			0.724	0.696
<1 years	18 (34.0)	14 (26.4)		
1–3 years	14 (26.4)	16 (30.2)		
≥ 3 years	21 (39.6)	23 (43.4)		
Medication class			0.052	0.819
SSRIs	40 (75.5)	41 (77.4)		
SNRIs	13 (24.5)	12 (22.6)		
Fluoxetine-equivalent dose, mg/day	24.8 ± 8.5	23.9 ± 9.1	0.479	0.633
Diabetes, yes	11 (20.8)	7 (13.2)	1.071	0.301
Hypertension, yes	16 (30.2)	9 (17.0)	2.565	0.109

-, Fisher’s exact test.

### 3.2 Primary outcome of patients

**Figure 2:**
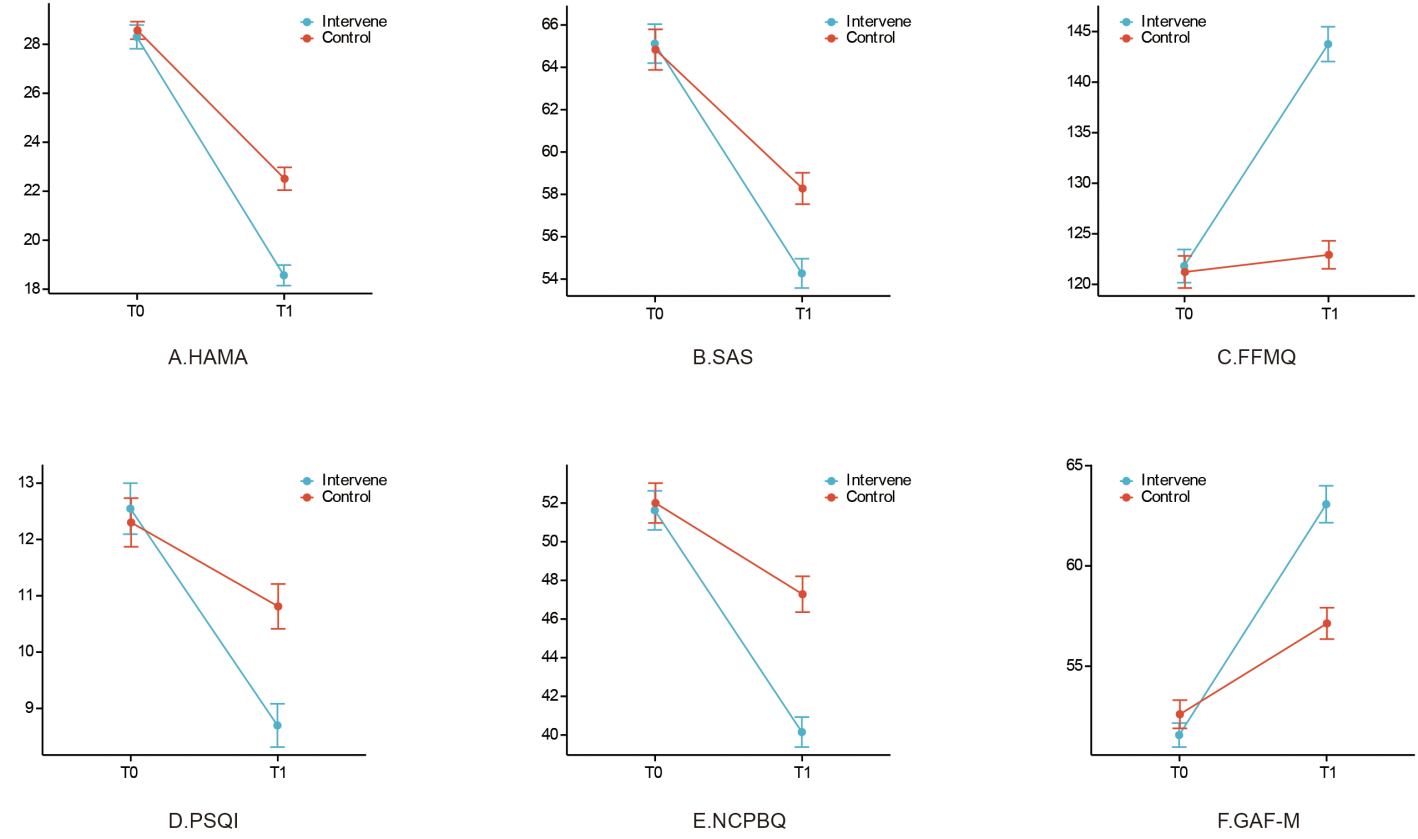
Trends in outcome. **(A)** HAMA: Hamilton Anxiety Rating Scale. **(B)** SAS: Self-Rating Anxiety Scale. **(C)** FFMQ: Five Facet Mindfulness Questionnaire. **(D)** PSQI: Pittsburgh Sleep Quality Index. **(E)** NCPBQ: Negative Cognitive Processing Bias Questionnaire. **(F)** GAF-M: Global Assessment of Functioning-Modified.

The baseline HAMA scores were comparable between the intervention group and the control group (28.3 vs. 28.6; *P* > 0.05) (**Table 2**). Following the 8-week intervention, patients in the intervention group achieved a significantly lower HAMA score (18.6) compared to the control group (22.5), with the between-group difference being statistically significant (*P* < 0.05). A LMM was fitted to further analyze the score trajectories. The results confirmed a significant group × time interaction (Estimate = -3.68, 95% CI [-5.38, -1.98], *P* < 0.05, Cohen’s d = 0.49), indicating that the reduction in anxiety severity over the study period was significantly greater in the intervention group than control group (**Table 3**). The differing trends in HAMA scores between the two groups across the assessment time points were presented in **Figure 2**.

**Table 2:** Comparison of outcome between groups at baseline and post-intervention.

Variables	Time	Intervention group (n=53)	Control group (n=53)	*t*	*P*
HAMA	T0	28.3 ± 3.5	28.6 ± 2.6	-0.438	0.662
	T1	18.6 ± 3.0	22.5 ± 3.4	-6.299	< 0.001
SAS	T0	65.1 ± 6.7	64.8 ± 7.0	0.213	0.832
	T1	54.3 ± 5.0	58.3 ± 5.4	-4.009	< 0.001
FFMQ	T0	121.8 ± 12.0	121.2 ± 11.6	0.256	0.798
	T1	143.8 ± 12.5	122.9 ± 10.1	9.436	< 0.001
PSQI	T0	12.6 ± 3.3	12.3 ± 3.1	0.392	0.696
	T1	8.7 ± 2.8	10.8 ± 2.9	-3.820	< 0.001
NCPBQ	T0	51.6 ± 7.4	52.0 ± 7.5	-0.262	0.794
	T1	40.2 ± 5.7	47.3 ± 6.7	-5.900	< 0.001
GAF-M	T0	51.6 ± 4.4	52.6 ± 5.1	-1.123	0.264
	T1	63.1 ± 6.7	57.1 ± 5.7	4.918	< 0.001

**Table 3:** Results of the LMM.

Variables	Estimate (95% CI)	*SE*	Cohen’s d	*t*	*P*
HAMA	-3.68 (-5.38, -1.98)	0.861	0.49	-4.273	< 0.001
SAS	-4.29 (-7.56, -1.03)	1.657	0.52	-2.591	0.010
FFMQ	20.25 (14.04, 26.45)	3.146	0.87	6.435	< 0.001
PSQI	-2.36 (-4.00, -0.71)	0.835	0.44	-2.825	0.005
NCPBQ	-6.75 (-10.43, -3.08)	1.865	0.61	-3.623	< 0.001
GAF-M	6.98 (4.01, 9.95)	1.507	0.68	4.632	< 0.001

### 3.3 Secondary outcomes of patients

Regarding secondary outcomes, baseline scores for all measures were comparable between groups (all *P* > 0.05). Post-intervention, the intervention group demonstrated significantly greater improvement across all domains compared to the control group. Specifically, the intervention group showed a greater reduction in SAS scores (54.3 vs. 58.3, *P* < 0.05; Estimate = -4.29, 95% CI [-7.56, -1.03], *P* < 0.05, Cohen’s d = 0.52) and NCPBQ scores (40.2 vs. 47.3, *P* < 0.05; Estimate = -6.75, 95% CI [-10.43, -3.08], *P* < 0.05, Cohen’s d = 0.61). Furthermore, the intervention group was associated with a significantly larger increase in FFMQ scores (143.8 vs. 122.9, *P* < 0.05; Estimate = 20.25, 95% CI [14.04, 26.45], *P* < 0.05, Cohen’s d = 0.87), greater improvement in PSQI scores (8.7 vs. 10.8, *P* < 0.05; Estimate = -2.36, 95% CI [-4.00, -0.71], *P* < 0.05, Cohen’s d = 0.44), and a more substantial enhancement in GAF-M scores (63.1 vs. 57.1, *P* < 0.05; Estimate = 6.98, 95% CI [4.01, 9.95], *P* < 0.05, Cohen’s d = 0.68). Detailed results were presented in **Tables 2**, **3**, and the change trends of all outcome measures were illustrated in **Figure 2**.

## 4 Discussion

The present randomized controlled trial provides robust empirical evidence supporting the adjunctive efficacy of a structured, 8-week MBI for patients with GAD concurrently receiving standard pharmacotherapy. Participants in the intervention group demonstrated significantly greater reductions in both clinician-rated and self-reported anxiety symptoms compared to the active control group receiving enhanced usual care. Beyond core symptom relief, the intervention conferred comprehensive multidimensional benefits, including marked improvements in trait mindfulness, subjective sleep quality, maladaptive cognitive patterns, and overall psychosocial functioning. These findings underscore the therapeutic potential of integrating mindfulness training into the conventional management paradigm for GAD to promote a more holistic recovery.

The significantly greater reduction in HAMA scores in the mindfulness group contributes important evidence to the existing literature on the adjunctive use of MBI alongside pharmacotherapy for GAD. This result aligns with meta-analytic reports of small to moderate effects of MBI on anxiety symptoms (11). The observed effect size for the primary outcome (Cohen’s d = 0.49) is considered moderate and clinically meaningful, as it represents a reduction of nearly 4 points more on the HAMA than in the control group. The effect size observed in the current trial may be considered more robust than estimates often derived from studies employing passive waitlist controls. This difference is likely attributable to our methodologically rigorous active control condition, which was carefully matched for time, attention, and group format. Such a design helps isolate the specific effects of mindfulness training from non-specific therapeutic factors, yielding a more conservative and clinically relevant efficacy estimate (10, 21). The significant group-by-time interaction from the LMM further confirms a superior trajectory of anxiety reduction in the intervention group. These results extend the evidence base by demonstrating a clinically meaningful benefit within a well-defined sample of pharmacotherapy-stabilized patients with moderate-to-severe GAD. This suggests that structured mindfulness training offers substantial additive value, potentially mitigating common limitations of pharmacotherapy alone, such as partial response or residual symptom persistence (12, 22). The integration of mindfulness skills, which target metacognitive awareness and emotional regulation, may operate synergistically with the primary neurochemical actions of medication, fostering a more comprehensive treatment response.

The most compelling contribution of this study is its demonstration of benefits that extend beyond symptomatic reduction to encompass functional recovery, cognitive change, and somatic well-being. The significant improvement in psychosocial functioning, as measured by the GAF-M, indicates that the intervention facilitated better life engagement and role performance. This finding provides valuable complementary evidence to a literature where many trials prioritize symptom scales over quantifiable assessments of daily functioning, despite the latter being a paramount treatment goal (11). Concurrently, the substantial increase in FFMQ scores, reflecting gains in skills such as non-judgmental awareness and cognitive decentering, offers a plausible mechanistic explanation for the broader therapeutic changes observed (23, 24). This cultivation of mindfulness skills likely underpinned the reduction in negative cognitive processing bias measured by the NCPBQ, suggesting participants learned to relate differently to patterns of worry and catastrophic thinking—a shift consistent with the theoretical model of mindfulness as a form of metacognitive training (25). Furthermore, the significant improvement in sleep quality, assessed via the PSQI, underscores the intervention’s holistic impact on a common and debilitating comorbidity in GAD that profoundly affects quality of life. While prior research has established links between mindfulness and sleep improvement, demonstrating this effect within a pharmacotherapy-stabilized GAD sample highlights its practical clinical relevance (26, 27). Collectively, these interconnected outcomes across functioning, cognition, and sleep depict an intervention that fosters broader psychological resilience and adaptive coping, advancing a more comprehensive model of recovery that integrates symptom alleviation with the enhancement of personal and social capacities.

The theoretical framework of MBI posits that fostering a metacognitive, decentered stance enables individuals to disrupt habitual patterns of cognitive-emotional reactivity (28). Our results, demonstrating mechanisms of cognitive bias reduction and mindfulness skill enhancement, provide empirical support for this model within the GAD context. This observed shift in cognitive processing aligns with neurobiological models of anxiety, which implicate hyperactivity in the amygdala and hypoactivity in prefrontal regulatory regions (29). Mindfulness training is theorized to strengthen top-down prefrontal control and attenuate amygdala reactivity, thereby enhancing emotion regulation and reducing the cognitive biases that perpetuate anxiety (30). The intervention appears to have targeted core maintaining factors of GAD, such as experiential avoidance and repetitive negative thinking, by promoting an accepting “being mode” over a reactive “doing mode” (31, 32). This shift may have reduced the distress associated with anxious thoughts and somatic sensations, thereby contributing to lower overall anxiety severity and improved functional capacity (33). The application of a LMM, which accounts for individual variability and within-subject correlation over time, provided a statistically robust analysis of these complex longitudinal changes, reinforcing the validity of the proposed mechanistic pathways (34).

The findings of this study have several important clinical implications. First, they suggest that integrating an 8-week MBI into routine pharmacotherapy can provide significant added value for patients with moderate-to-severe GAD, addressing residual symptoms and improving functional outcomes. Second, the structured, manualized nature of the intervention makes it suitable for implementation in mental health settings, potentially by trained nurses or psychologists, after appropriate training. Third, while a formal cost-effectiveness analysis was beyond the scope of this trial, the group-based format is relatively resource-efficient compared to individual psychotherapy, suggesting potential for scalability. Healthcare systems could consider incorporating such evidence-based mindfulness programs into clinical pathways for anxiety disorders to promote more holistic recovery. Despite these positive findings, several limitations of the current study must be acknowledged. First, the follow-up period was restricted to the immediate post-intervention assessment. Consequently, the long-term sustainability of the observed clinical gains, including the prevention of symptom relapse, remains unknown and warrants investigation through extended follow-up studies. Second, while the sample size was adequate for detecting the primary outcome, a larger, multi-center trial would enhance the generalizability of the findings across diverse healthcare settings and patient demographics. Third, although medication stability was a prerequisite and actively monitored, the specific types and dosages of pharmacotherapy were not standardized, introducing a potential source of heterogeneity. Future research could employ a more stratified design to explore potential interactions between specific drug classes and mindfulness training.

## 5 Conclusion

In conclusion, this randomized controlled trial provides evidence that an 8-week MBI, as an adjunct to pharmacotherapy, is effective in not only reducing anxiety symptoms but also enhancing mindfulness skills, improving sleep quality, ameliorating negative cognitive biases, and boosting psychosocial functioning in patients with GAD. These results advocate for the integration of such evidence-based mindfulness programs into routine clinical pathways for anxiety disorders to promote a more holistic and functional recovery. Future research should focus on examining the durability of benefits, conducting cost-effectiveness analyses, elucidating neurobiological mechanisms, and refining protocols for optimal implementation in real-world clinical settings.

## Data Availability

The original contributions presented in the study are included in the article/supplementary material. Further inquiries can be directed to the corresponding author.
